# Objective measures of smoking and caffeine intake and the risk of adverse pregnancy outcomes

**DOI:** 10.1093/ije/dyad123

**Published:** 2023-09-28

**Authors:** Roshan J Selvaratnam, Ulla Sovio, Emma Cook, Francesca Gaccioli, D Stephen Charnock-Jones, Gordon C S Smith

**Affiliations:** The Ritchie Centre, Department of Obstetrics and Gynaecology, Monash University, VIC, Melbourne, Australia; Department of Obstetrics and Gynaecology, University of Cambridge, Cambridge, UK; Centre for Trophoblast Research, Department of Physiology, Development and Neuroscience, University of Cambridge, Cambridge, UK; Department of Obstetrics and Gynaecology, University of Cambridge, Cambridge, UK; Department of Obstetrics and Gynaecology, University of Cambridge, Cambridge, UK; Centre for Trophoblast Research, Department of Physiology, Development and Neuroscience, University of Cambridge, Cambridge, UK; Department of Obstetrics and Gynaecology, University of Cambridge, Cambridge, UK; Centre for Trophoblast Research, Department of Physiology, Development and Neuroscience, University of Cambridge, Cambridge, UK; Department of Obstetrics and Gynaecology, University of Cambridge, Cambridge, UK; Centre for Trophoblast Research, Department of Physiology, Development and Neuroscience, University of Cambridge, Cambridge, UK

**Keywords:** Smoking, caffeine, paraxanthine, cotinine, pre-eclampsia, fetal growth restriction, pre-term birth, gestational diabetes, birthweight

## Abstract

**Background:**

In pregnancy, women are encouraged to cease smoking and limit caffeine intake. We employed objective definitions of smoking and caffeine exposure to assess their association with adverse outcomes.

**Methods:**

We conducted a case cohort study within the Pregnancy Outcome Prediction study to analyse maternal serum metabolomics in samples from 12, 20, 28 and 36 weeks of gestational age. Objective smoking status was defined based on detectable cotinine levels at each time point and objective caffeine exposure was based on tertiles of paraxanthine levels at each time point. We used logistic and linear regression to examine the association between cotinine, paraxanthine and the risk of pre-eclampsia, spontaneous pre-term birth (sPTB), fetal growth restriction (FGR), gestational diabetes mellitus and birthweight.

**Results:**

There were 914 and 915 women in the smoking and caffeine analyses, respectively. Compared with no exposure to smoking, consistent exposure to smoking was associated with an increased risk of sPTB [adjusted odds ratio (aOR) = 2.58, 95% CI: 1.14 to 5.85)] and FGR (aOR = 4.07, 95% CI: 2.14 to 7.74) and lower birthweight (β = –387 g, 95% CI: –622 g to –153 g). On univariate analysis, consistently high levels of paraxanthine were associated with an increased risk of FGR but that association attenuated when adjusting for maternal characteristics and objective—but not self-reported—smoking status.

**Conclusions:**

Based on objective data, consistent exposure to smoking throughout pregnancy was strongly associated with sPTB and FGR. High levels of paraxanthine were not independently associated with any of the studied outcomes and were confounded by smoking.

Key Messages

- Most studies reporting the association between smoking and caffeine intake during pregnancy and adverse outcomes are based on self-reported subjective data.
- We conducted a case cohort study to examine the associations between objective measures of smoking and caffeine in pregnancy and the risk of adverse outcomes.
- Compared with other studies, we observed stronger associations between consistent smoking exposure and fetal growth restriction [adjusted odds ratio (aOR) = 4.07], spontaneous pre-term birth (aOR = 2.58) and lower birthweights (β = –387 g), whereas there was no evidence of a reduced risk of pre-eclampsia.
- Caffeine exposure was not independently associated with an increased risk of adverse pregnancy outcomes.
- Smoking confounded the associations between paraxanthine and adverse pregnancy outcomes but adjustment using self-reported smoking was associated with residual confounding through misclassification of smoking status.

## Introduction

In pregnancy, limiting caffeine intake and ceasing smoking are widely recommended. These recommendations come from decades of research that have assessed their relationship with pregnancy complications. For instance, smoking during pregnancy is associated with an increased risk of fetal growth restriction (FGR),^1^ spontaneous pre-term birth^2^ and low birthweight^3^^,^^4^ but a reduced risk of pre-eclampsia (PE).^5^ The paradoxical effect on PE risk is thought to be related to the effects of smoking on pro-angiogenic proteins, such as increasing placental growth factor (PlGF), and anti-angiogenic proteins, such as decreasing soluble fms-like tyrosine kinase 1 (sFlt-1),^6^ though data are inconclusive. On the other hand, high caffeine intake has been shown to be associated with lower birthweights,^7^^,^^8^ smaller neonatal anthropometric measurements^9^ and possibly FGR.^10^^,^^11^ This is concerning because caffeine is the most-used xenobiotic in pregnancy, found in coffee, tea, chocolate, energy drinks, soft drinks and certain medications, with the potential to affect a large number of pregnancies.

The challenge with interpreting studies reporting associations between smoking, caffeine and adverse pregnancy outcomes is that many have relied on self-reported data to estimate exposure, which is prone to recall bias and under-reporting^12^^,^^13^ and may not reflect accurately the quantity that enters the maternal circulation. A more objective measure of exposure is to directly quantify the primary metabolites of tobacco and caffeine in maternal body fluids. For example, the primary metabolite of nicotine is cotinine, which is a sensitive marker of tobacco exposure that can be detected in blood, urine and saliva.^14^ The primary metabolite of caffeine is paraxanthine, which accounts for 80% of caffeine metabolism and is both less sensitive to recent intake and more stable throughout the day.^15^^,^^16^ Using these metabolites to estimate the risk of adverse outcomes may be superior to using self-reported measures, particularly if assessed at multiple time points throughout the pregnancy.

In this study, we used objective measures of smoking and caffeine intake, i.e. maternal serum cotinine and paraxanthine levels at different time points during gestation, to assess their association with adverse pregnancy outcomes.

## Methods

### Study design

We employed a case cohort design from the Pregnancy Outcome Prediction (POP) study, which has been described in detail elsewhere.^17^^,^^18^ In brief, a total of 4212 nulliparous women attending the Rosie Hospital, Cambridge, UK between 2008 and 2012 were recruited at the time of their dating ultrasound and followed until delivery. The case cohort included 638 women who had at least one measured adverse pregnancy outcome [PE, spontaneous pre-term delivery (sPTB), FGR or gestational diabetes mellitus (GDM)] and a random sample of 323 women, of whom 277 did not experience any of the above adverse outcomes (flow diagram in Supplementary Figure S1, available as Supplementary data at *IJE* online). Blood samples were collected at four time points—at ∼12, 20, 28 and 36 weeks of gestational age (wkGA)—but in the case of GDM we only analysed samples up to 28 wkGA as this is when the diagnosis is typically made. Pregnancy outcome data were obtained by individual review of paper-based hospital records and relevant electronic databases. For this study, women with only one of the four possible blood samples were excluded (*n* = 8) (see number of missing samples in Supplementary Figure S1 and Supplementary Table S1, available as Supplementary data at *IJE* online). Cambridgeshire 2 Research Ethics Committee gave ethical approval for the study (reference number 07/H0308/163) and all participants provided written informed consent.

### Outcomes

The primary outcomes were PE, sPTB, FGR and GDM. PE was defined in accordance with the American College of Obstetricians and Gynecologists (ACOG) 2013 Guidelines^19^ and included all women with pre-term PE, severe term PE and non-severe non-superimposed term PE.^18^ Non-severe superimposed term PE was not included in the analysis of PE. sPTB was defined as birth before 37 wkGA in the absence of induction of labour or pre-labour caesarean section. Medically indicated pre-term births within the random sub-cohort were excluded from the analysis of sPTB. FGR was defined as customized birthweight below the 10th percentile if delivery was before 37 wkGA.^20^ If delivery was at or after 37 wkGA, FGR was defined as a birthweight below the 3rd percentile or birthweight between the 3rd and 10th percentiles combined with the lowest decile of fetal abdominal growth velocity.^21^^,^^22^ GDM was defined using the criteria adapted from the World Health Organization from 2008 to 2010 and the criteria adapted from the International Association of Diabetes and Pregnancy Study Groups from 2011 onward.^23^ A secondary outcome was birthweight, which was assessed in grams (crude), customized *Z*-score and customized percentile.^20^

### Biochemical analyses

Serum metabolites were measured by Metabolon using non-targeted Ultrahigh Performance Liquid Chromatography–Tandem Mass Spectroscopy, as previously described.^18^^,^^21^ Cotinine, a tobacco metabolite, and paraxanthine, a caffeine metabolite, were assessed. Maternal blood samples with undetectable values of cotinine or paraxanthine were assumed to be the result of falling below the detection sensitivity and, thus, were imputed with the minimum detection value based on each metabolite. To adjust for instrument batch effects for each run day, the raw ion counts for each metabolite were divided by the median value for the run day and so metabolites were expressed as multiples of the median (MoM). Maternal serum levels of sFlt-1 and PlGF were measured using Roche Elecsys assays on the electrochemiluminescence immunoassay platform, Cobas e411 (Roche Diagnostics), as previously described.^24^

### Objective measures

Global measures of objective smoking status and objective caffeine exposure across the entire pregnancy were created from metabolite levels at 12, 20, 28 and 36 wkGA. Women with only one measurement were excluded from the study (*n* = 8). Based on cotinine MoMs at each time point, no exposure to smoking was defined by cotinine being undetectable at every time point where samples were available. Consistent exposure to smoking was defined as cotinine being detectable at every time point where samples were available. All other women were considered to have some (inconsistent) exposure to smoking. Women who self-reported using nicotine replacement therapy (*n* = 1) were excluded from smoking analyses as serum tobacco metabolites may not reflect true smoking exposure. Objective smoking status was compared with self-reported smoking status based on a questionnaire given to women at 20 wkGA. Objective caffeine exposure was based on paraxanthine MoMs in the random sub-cohort at 12, 20, 28 and 36 wkGA. Tertile cut-offs were calculated at each time point and applied to the entire case cohort population. If a woman did not have a maternal blood sample at a given time point, no tertile was calculated. Low levels of paraxanthine throughout pregnancy were defined as a woman who was in the lowest tertile at every measurement and were used as a proxy for low caffeine intake during pregnancy. High levels of paraxanthine throughout pregnancy were defined as a woman who was in the highest tertile at every measurement and were used as a proxy for high caffeine intake during pregnancy. All other women were defined as having moderate levels of paraxanthine throughout pregnancy.

### Statistical analysis

The association between the primary outcomes and the defined measures of objective smoking status and objective caffeine exposure were analysed first using unadjusted logistic regression. Cases with the given outcome of interest were compared with members of the random sub-cohort who did not experience the given outcome. Logistic regression was then repeated, adjusting for potential confounders: maternal height, maternal age, maternal body mass index, marital status, age at stopping full-time education and socio-economic deprivation in quartiles. Maternal age was defined as age at recruitment. The weight measurement used in the body mass index calculation was performed at the 12-wkGA visit. All other maternal characteristics were defined by self-report at the 20-wkGA questionnaire, from examination of the clinical case record or from linkage to the electronic databases of the hospital. Socio-economic status was quantified using the Index of Multiple Deprivation 2007,^25^ which is based on census data from the area of the mother’s postcode. Deprivation quartiles were calculated referent to the POP study. Due to small numbers of non-White women, ethnicity was not added as a covariate in multivariable models but was addressed in a sensitivity analysis. Weight gain during pregnancy was also assessed as a covariate in sensitivity analysis. Weight gain was defined as the difference in maternal weight between 12 and 36 wkGA and transformed into a *Z*-score. When examining pre-term deliveries in any analysis and any participants in the analysis of sPTB and GDM, weight gain was defined as the difference in maternal weight between 12 and 28 wkGA and then transformed into a *Z*-score. The frequency of missing values of the covariates was small and so they were imputed using the mode for categorical variables and mean for continuous variables. For regression analysis relating to caffeine exposure, objective smoking status was added as a covariate due to the correlation between paraxanthine and smoking.^26^ Crude birthweight and birthweight Z-scores were examined using univariable and multivariable linear regression. The whole case cohort was studied by weighting the non-cases of the random sub-cohort by the inverse of the sampling fraction. The sampling fraction was calculated as per our previous publications (4177/325 = 12.85).^18^ Quantile regression using the median was performed to assess the birthweight percentile because of its underlying uniform distribution.

In addition, given previous literature suggesting that smoking may reduce the risk of PE via effects on pro-angiogenic and anti-angiogenic proteins,^6^^,^^27^^,^^28^ we performed multivariable linear regression using the random sub-cohort to assess the relationship between objective smoking status and maternal sFlt-1 and PlGF levels measured at ∼12, 20, 28 and 36 wkGA. sFlt-1 and PlGF levels were expressed as MoM, adjusted for the exact gestational age and maternal weight at the time the sample was taken, and storage time at sample measurement, and then log-transformed and converted into *Z*-scores, referent to the whole POP study cohort. We used the chi-square test of independence to examine whether there was an association between objective smoking status and objective caffeine exposure. We also calculated Spearman’s correlation coefficients between cotinine and paraxanthine MoMs at 12, 20, 28 and 36 wkGA. To assess whether adjusting for objective smoking status was superior to self-reported smoking in multivariable logistic regression models for paraxanthine, we examined the association between paraxanthine tertiles at 12, 20, 28 and 36 wkGA, separately, and FGR. Paraxanthine tertiles at different time points were assumed to be independent of each other. FGR was chosen for this analysis because other studies have shown an increased risk in caffeine consumers.^10^^,^^11^

## Results

After exclusions, there were a total of 914 patients for the smoking analyses and 915 patients for the caffeine analyses. Of the 914 patients in the smoking analyses, 718 (78.6%), 107 (11.7%) and 89 (9.7%) were classified as having no exposure, some exposure and consistent exposure to smoking throughout pregnancy, respectively. Of the 915 patients in the caffeine analyses, 117 (12.8%), 677 (74.0%) and 121 (13.2%) were classified as having low, moderate and high levels of paraxanthine throughout pregnancy, respectively. Table 1 shows the maternal characteristics of both populations and the rate of each studied adverse pregnancy outcome.

**Table 1.** Maternal characteristics and primary outcomes by objective smoking status and objective caffeine exposure

	**Objective smoking status (*n*=914)** ^a^			**Objective caffeine exposure (*n*=915)** ^a^		
	No exposure to smoking throughout pregnancy (*n*=718)	Some exposure to smoking throughout pregnancy (*n*=107)	Consistent exposure to smoking throughout pregnancy (*n*=89)	Low levels of paraxanthine throughout pregnancy (*n*=117)	Moderate levels of paraxanthine throughout pregnancy (*n*=677)	High levels of paraxanthine throughout pregnancy (*n*=121)
Age (years)	31 (28–34)	30 (25–33)	26 (22–31)	32 (29–35)	30 (26–33)	31 (27–35)
Age at stopping full-time education (years)	21 (18–23)	19 (16–22)	17 (16–18)	21 (18–23)	21 (18–23)	19 (17–22)
Missing	15 (2.1%)	6 (5.6%)	3 (3.4%)	6 (5.1%)	15 (2.2%)	3 (2.5%)
Height (cm)	165 (160–168)	165 (160–168)	163 (160–168)	165 (161–168)	164 (160–168)	164 (160–169)
Body mass index (kg/m^2^)	25 (22–28)	26 (23–30)	25 (22–30)	26 (23–29)	25 (22–29)	26 (23–29)
Missing	1 (0.1%)	0	0	1 (0.9%)	0	0
Married	533 (74.2%)	69 (64.5%)	35 (39.3%)	89 (76.1%)	478 (70.6%)	70 (57.9%)
Deprivation quartile						
1 (least deprived)	185 (25.8%)	22 (20.6%)	8 (9.0%)	24 (20.5%)	164 (24.2%)	27 (22.3%)
2	173 (24.1%)	28 (26.2%)	21 (23.6%)	28 (23.9%)	160 (23.6%)	34 (28.1%)
3	173 (24.1%)	32 (29.9%)	21 (23.6%)	23 (19.7%)	167 (24.7%)	36 (29.8%)
4 (most deprived)	162 (22.6%)	21 (19.6%)	33 (37.1%)	38 (32.5%)	162 (23.9%)	17 (14.1%)
Missing	25 (3.5%)	4 (3.7%)	6 (6.7%)	4 (3.4%)	24 (3.6%)	7 (5.8%)
White ethnicity	665 (92.6%)	98 (91.6%)	88 (98.9%)	103 (88.0%)	631 (93.2%)	118 (97.5%)
Missing	14 (2.0%)	1 (0.9%)	0	3 (2.6%)	12 (1.8%)	0
Primary outcomes^b^						
PE	150	28	12	26	140	24
sPTB	86	11	14	13	82	16
FGR	146	27	42	20	159	36
GDM	148	25	12	34	127	24

FGR, fetal growth restriction; GDM, gestational diabetes mellitus; IQR, interquartile range; PE, pre-eclampsia; sPTB, spontaneous pre-term birth.

a Data are expressed as median (IQR) or *n* (%) as appropriate, with the number of missing values shown below each characteristic. Where there is no missing category, data were complete.

b Only the number of cases of the given outcome are presented.

Of the 89 patients with consistent exposure to smoking throughout pregnancy, only 61 (68.5%) self-reported as currently smoking (Table 2). There was an association between objective caffeine exposure and objective smoking status (*P *<* *0.001), with 10.6% (*n* = 76) of women with no exposure to smoking throughout pregnancy having high levels of paraxanthine throughout pregnancy but 31.5% (*n* = 28) of women with consistent exposure to smoking throughout pregnancy having high levels of paraxanthine throughout pregnancy (Table 2). There was a correlation between cotinine and paraxanthine MoMs at 12, 20, 28 and 36 wkGA (Spearman’s correlation coefficients: 0.17, 0.19, 0.13 and 0.28, respectively, all *P *<* *0.001).

**Table 2.** Cross tabulation of objective smoking status, self-reported smoking status and objective caffeine exposure

	**Objective smoking status** ^a^			
	No exposure to smoking throughout pregnancy	Some exposure to smoking throughout pregnancy	Consistent exposure to smoking throughout pregnancy	Total
**Self-reported smoking status**				
Currently smoking	0	5 (4.7%)	61 (68.5%)	66
Never smoked	468 (65.2%)	50 (46.7%)	3 (3.4%)	521
Quit smoking during pregnancy	33 (4.6%)	18 (16.8%)	21 (23.6%)	72
Quit smoking before pregnancy	217 (30.2%)	34 (31.8%)	4 (4.5%)	255
Total	718	107	89	914
**Objective caffeine exposure**				
Low levels of paraxanthine throughout pregnancy	102 (14.2%)	10 (9.4%)	5 (5.6%)	718
Moderate levels of paraxanthine throughout pregnancy	540 (75.2%)	80 (74.8%)	56 (62.9%)	107
High levels of paraxanthine throughout pregnancy	76 (10.6%)	28 (31.5%)	28 (31.5%)	89
Total	117	676	121	914

a Data are expressed as *n* (column %) for cross tabulation between objective smoking status and self-reported smoking status and as *n* (row %) for cross tabulation between objective smoking status and objective caffeine exposure. Self-reported smoking status was based on a questionnaire given to women at 20 weeks of gestational age.

Table 3 shows the unadjusted and adjusted odds ratios (aORs) between objective smoking status and adverse pregnancy outcomes. Compared with no exposure to smoking throughout pregnancy, consistent exposure to smoking throughout pregnancy was associated with an increased risk of sPTB (aOR = 2.58, 95% CI: 1.14 to 5.85) and FGR (aOR = 4.07, 95% CI: 2.14 to 7.74) and was associated with lower birthweight (β = –387 g, 95% CI: –622 g to –153 g), birthweight Z-score (β = –0.61, 95% CI: –0.95 to –0.27) and median birthweight percentile (β = –24, 95% CI: –39 to –8). It did not reduce the risk of PE (aOR = 0.90, 95% CI: 0.39 to 2.05). Some exposure to smoking throughout pregnancy did not increase the risk of PE, sPTB, FGR or GDM, but was associated with lower birthweight (β = –169 g, 95% CI: –292 g to –46 g) and birthweight Z-score (β = –0.30, 95% CI: –0.53 to –0.07) compared with no exposure to smoking throughout pregnancy.

**Table 3.** Association between objective smoking status and adverse pregnancy outcomes

Outcome	Objective smoking status							
	**Some exposure to smoking throughout pregnancy** ^a^				**Consistent exposure to smoking throughout pregnancy** ^a^			
	**Univariable analysis** ^a^		**Multivariable analysis** ^a,^ ^e^		**Univariable analysis** ^a^		**Multivariable analysis** ^a,^ ^e^	
	OR (95% CI)	*P*	aOR (95% CI)	*P*	OR (95% CI)	*P*	aOR (95% CI)	*P*
PE	1.46 (0.85, 2.53)	0.17	1.35 (0.76, 2.42)	0.31	1.06 (0.50, 2.24)	0.89	0.90 (0.39, 2.05)	0.80
sPTB	1.00 (0.48, 2.06)	0.99	1.02 (0.49, 2.15)	0.95	2.09 (1.01, 4.32)	0.05	2.58 (1.14, 5.85)	0.02
FGR	1.50 (0.86, 2.61)	0.15	1.44 (0.82, 2.56)	0.21	3.80 (2.13, 6.78)	< 0.001	4.07 (2.14, 7.74)	<0.001
GDM	1.36 (0.77, 2.38)	0.29	1.33 (0.72, 2.46)	0.36	0.95 (0.46, 1.97)	0.88	1.06 (0.47, 2.43)	0.89

	**β coefficient (95% CI)**	***P***	**β coefficient (95% CI)**	***P***	**β coefficient (95% CI)**	***P***	**β coefficient (95% CI)**	***P***

Birthweight (g)^b^	–154 (–282, –26)	0.02	–169 (–292, –46)	0.01	–441 (–674, –207)	<0.001	–387 (–622, –153)	0.001
Birthweight Z-score^b,^^c^	–0.33 (–0.56, –0.09)	0.01	–0.30 (–0.53, –0.07)	0.01	–0.70 (–1.01, –0.39)	<0.001	–0.61 (–0.95, –0.27)	<0.001
Birthweight percentile^b,^^d^	–11 (–27, 5)	0.17	–8 (–18, 3)	0.15	–29 (–47, –11)	0.002	–24 (–39, –8)	0.003

The numbers of cases and non-cases from the random sub-cohort included in the analyses of the binary outcomes were 190 PE cases and 302 non-cases, 111 sPTB cases and 310 non-cases, 215 FGR cases and 301 non-cases, and 185 GDM cases and 311 non-cases.

aOR, adjusted odds ratio; FGR, fetal growth restriction; GDM, gestational diabetes mellitus; OR, odds ratio; PE, pre-eclampsia; sPTB, spontaneous pre-term birth.

a Reference group is women with no exposure to smoking throughout pregnancy.

b For birthweight regression analyses, the entire case cohort (*n*=914) was studied by weighting the non-cases of the random sub-cohort by the inverse of the sampling fraction.

c For the birthweight *Z*-scores, β coefficients from linear regression analyses were given per 1-SD increase in the birthweight percentile.

d For the birthweight percentile, quantile regression analyses using the median were performed.

e Adjusting for maternal height, maternal age, maternal body mass index, marital status, maternal age at stopping full-time education and deprivation.

**Figure 1.**
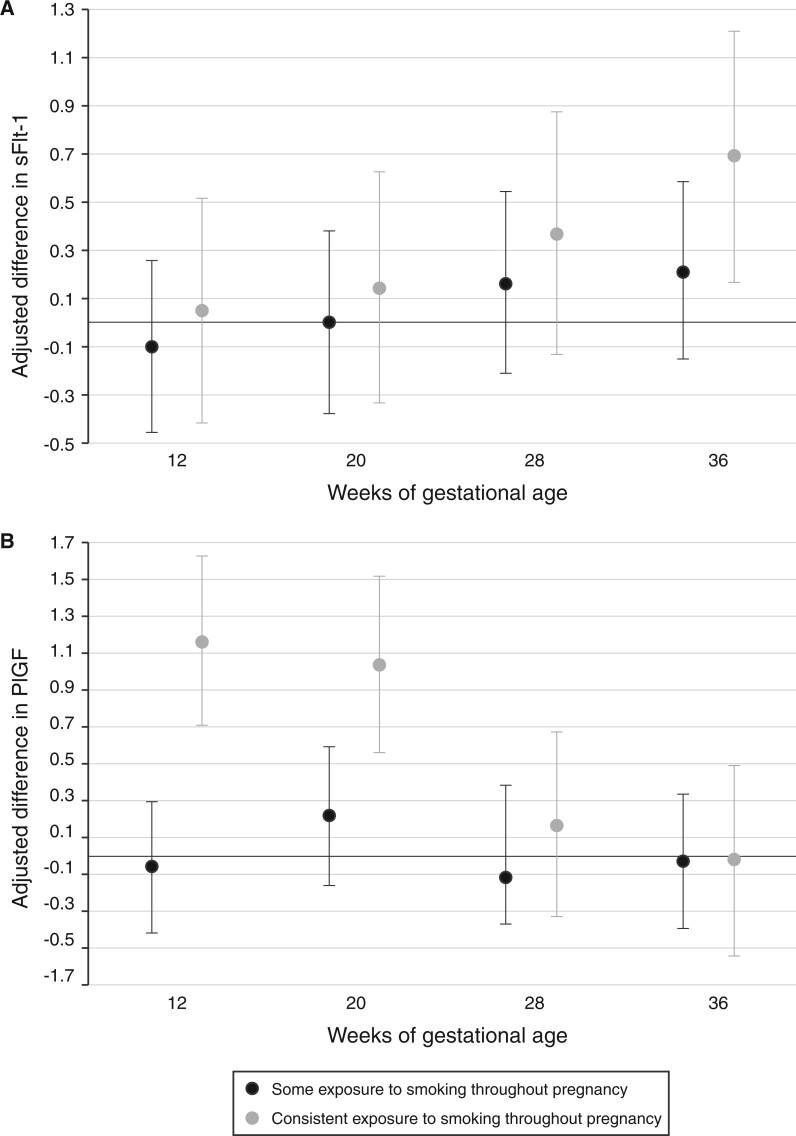
Adjusted difference (with 95% CI) in maternal sFlt-1 (A) and PlGF (B) values by objective smoking status at 12, 20, 28 and 36 wkGA, referent to no exposure to smoking throughout pregnancy. sFlt-1, soluble fms-like tyrosine kinase; PlGF, placental growth factor; wkGA, weeks of gestational age; MoM, multiples of the median. Maternal sFlt-1 and PlGF levels have been expressed as MoM, adjusted for gestational age, maternal weight and storage time at measurement and then log-transformed and converted into *Z*-scores, referent to the whole Pregnancy Outcome Prediction study cohort. Multivariable linear regression analyses were performed on the random sub-cohort. The reference group is women with no exposure to smoking throughout pregnancy. β coefficients were given per 1-SD increase in sFlt-1 or PlGF. The multivariable analyses were adjusted for maternal height, maternal age, maternal body mass index, marital status, maternal age at stopping full-time education and deprivation

In the random sub-cohort, sFlt-1 levels were more than half an SD higher at 36 wkGA (β = 0.69, 95% CI: 0.17 to 1.20) and PlGF levels were >1 SD higher at 12 wkGA (β = 1.17, 95% CI: 0.72 to 1.61) and 20 wkGA (β = 1.04, 95% CI: 0.59 to 1.48) in women who had consistent exposure to smoking throughout pregnancy compared with no exposure to smoking throughout pregnancy (Figure 1).

Table 4 presents the unadjusted and adjusted odds of adverse pregnancy outcomes in relation to objective caffeine exposure. Compared with women with low levels of paraxanthine, those with moderate levels of paraxanthine had a reduced risk of GDM (aOR = 0.57, 95% CI: 0.32 to 1.00). There was no increased risk of PE, sPTB, FGR or lower birthweight associated with moderate or high levels of paraxanthine throughout pregnancy after adjustments.

**Table 4.** Association between objective caffeine exposure and adverse pregnancy outcomes

Outcome	Objective caffeine exposure							
	**Moderate levels of paraxanthine throughout pregnancy** ^a^				**High levels of paraxanthine throughout pregnancy** ^a^			
	**Univariable analysis** ^a^		**Multivariable analysis** ^a,^ ^e^		**Univariable analysis** ^a^		**Multivariable analysis** ^a,^ ^e^	
	OR (95% CI)	*P*	aOR (95% CI)	*P*	OR (95% CI)	*P*	aOR (95% CI)	*P*
PE	0.78 (0.45, 1.35)	0.38	0.76 (0.42, 1.37)	0.36	0.92 (0.44, 1.92)	0.83	0.90 (0.41, 1.97)	0.80
sPTB	0.86 (0.43, 1.71)	0.67	0.83 (0.41, 1.69)	0.60	1.13 (0.47, 2.70)	0.79	0.83 (0.33, 2.10)	0.69
FGR	1.08 (0.60, 1.96)	0.79	0.94 (0.51, 1.75)	0.85	1.69 (0.82, 3.51)	0.16	1.19 (0.55, 2.59)	0.66
GDM	0.54 (0.32, 0.91)	0.02	0.57 (0.32, 1.00)	0.05	0.69 (0.34, 1.38)	0.29	0.60 (0.27, 1.31)	0.20

	**β coefficient (95% CI)**	***P***	**β coefficient (95% CI)**	***P***	**β coefficient (95% CI)**	***P***	**β coefficient (95% CI)**	***P***

Birthweight (g)^b^	–99 (–258, 59)	0.22	–52 (–203, 100)	0.50	–124 (–370, 122)	0.32	0 (–220, 220)	>0.99
Birthweight *Z*-score^b,^^c^	–0.29 (–0.58, –0.01)	0.05	–0.21 (–0.50, 0.08)	0.15	–0.31 (–0.68, 0.06)	0.10	–0.07 (–0.44, 0.29)	0.69
Birthweight percentile^b,^^d^	–8 (–26, 10)	0.37	–3 (–19, 14)	0.75	–4 (–22, 14)	0.66	7 (–13, 26)	0.49

The numbers of cases and non-cases from the random sub-cohort included in the analyses of the binary outcomes were 190 PE cases and 303 non-cases, 111 sPTB cases and 311 non-cases, 215 FGR cases and 301 non-cases, and 185 GDM cases and 312 non-cases.

aOR, adjusted odds ratio; FGR, fetal growth restriction; GDM, gestational diabetes mellitus; OR, odds ratio; PE, pre-eclampsia; sPTB, spontaneous pre-term birth.

a Reference group is women with low levels of paraxanthine throughout pregnancy.

b For the birthweight regression analyses, the entire case cohort (*n*=915) was studied by weighting the non-cases of the random sub-cohort by the inverse of the sampling fraction.

c For the birthweight *Z*-score, β coefficients from linear regression analyses were given per 1-SD increase in the birthweight percentile.

d For the birthweight percentile, quantile regression analyses using the median were performed.

e Adjusting for objective smoking status, maternal height, maternal age, maternal body mass index, marital status, maternal age at stopping full-time education and deprivation.

Sensitivity analyses that added ethnicity to multivariable models did not materially change the aORs or regression coefficients presented in Tables 3 and 4. Weight gain during pregnancy was also added to multivariable models and there was no material change in our results (Supplementary Tables S2 and S3, available as Supplementary data at *IJE* online). However, weight gain during pregnancy may lie on the causal pathway from the exposure to the weight-related outcomes.

Table 5 presents the association between paraxanthine tertile at 12, 20, 28 and 36 wkGA, separately, and FGR. There was a positive association between the paraxanthine tertile and FGR at 12, 28 and 36 wkGA in univariate analysis. However, all of these associations were attenuated following adjustment for maternal characteristics and objective smoking status. The degree of attenuation was lower when the associations were adjusted for maternal characteristics and self-reported smoking status.

**Table 5.** Unadjusted and adjusted odds ratios of fetal growth restriction for paraxanthine tertiles at 12, 20, 28 and 36 weeks of gestational age

	Univariable analyses		**Model A** ^a^		**Model B** ^b^		**Model C** ^c^	
	**Odds ratio (95% CI)** ^d^	*P*	**Odds ratio (95% CI)** ^d^	*P*	**Odds ratio (95% CI)** ^d^	*P*	**Odds ratio (95% CI)** ^d^	*P*
Paraxanthine at 12 wkGA	1.33 (1.07, 1.67)	0.01	1.31 (1.04, 1.64)	0.02	1.23 (0.97, 1.55)	0.09	1.27 (1.00, 1.60)	0.05
Paraxanthine at 20 wkGA	1.21 (0.97, 1.51)	0.09	1.16 (0.92, 1.46)	0.20	1.04 (0.82, 1.32)	0.74	1.08 (0.86, 1.37)	0.51
Paraxanthine at 28 wkGA	1.31 (1.04, 1.64)	0.02	1.29 (1.02, 1.63)	0.03	1.21 (0.96, 1.54)	0.11	1.25 (0.99, 1.58)	0.07
Paraxanthine at 36 wkGA	1.47 (1.15, 1.87)	0.002	1.43 (1.12, 1.84)	0.005	1.28 (0.98, 1.65)	0.07	1.37 (1.06, 1.76)	0.02

	**Odds ratio (95% CI)** ^e^	***P***	**Odds ratio (95% CI)** ^e^	***P***	**Odds ratio (95% CI)** ^e^	***P***	**Odds ratio (95% CI)** ^e^	***P***

Paraxanthine at 12 wkGA								
Tertile 1	Ref.		Ref.		Ref.		Ref.	
Tertile 2	1.42 (0.90, 2.23)	0.13	1.37 (0.86, 2.18)	0.19	1.32 (0.82, 2.12)	0.25	1.36 (0.85, 2.18)	0.20
Tertile 3	1.79 (1.14, 2.79)	0.01	1.72 (1.08, 2.72)	0.02	1.52 (0.95, 2.43)	0.08	1.61 (1.01, 2.57)	0.05
Paraxanthine at 20 wkGA								
Tertile 1	Ref.		Ref.		Ref.		Ref.	
Tertile 2	1.23 (0.79, 1.93)	0.36	1.18 (0.75, 1.88)	0.47	1.10 (0.68, 1.76)	0.70	1.11 (0.70, 1.77)	0.66
Tertile 3	1.47 (0.94, 2.29)	0.09	1.35 (0.85, 2.13)	0.20	1.09 (0.68, 1.75)	0.73	1.17 (0.73, 1.88)	0.51
Paraxanthine at 28 wkGA								
Tertile 1	Ref.		Ref.		Ref.		Ref.	
Tertile 2	1.45 (0.91, 2.30)	0.12	1.39 (0.86, 2.23)	0.18	1.32 (0.82, 2.14)	0.26	1.36 (0.84, 2.20)	0.21
Tertile 3	1.73 (1.09, 2.72)	0.02	1.67 (1.05, 2.68)	0.03	1.49 (0.92, 2.40)	0.11	1.57 (0.98, 2.53)	0.06
Paraxanthine at 36 wkGA								
Tertile 1	Ref.		Ref.		Ref.		Ref.	
Tertile 2	1.55 (0.93, 2.57)	0.09	1.43 (0.85, 2.41)	0.18	1.36 (0.80, 2.31)	0.26	1.37 (0.81, 2.33)	0.24
Tertile 3	2.17 (1.33, 3.54)	0.002	2.06 (1.24, 3.40)	0.005	1.64 (0.97, 2.77)	0.06	1.87 (1.12, 3.12)	0.02

FGR, fetal growth restriction; Ref., reference; wkGA, weeks of gestational age.

The number of FGR cases and non-cases from the random sub-cohort included in the analyses were 211 FGR cases and 281 non-cases at 12 wkGA, 210 FGR cases and 287 non-cases at 20 wkGA, 201 FGR cases and 286 non-cases at 28 wkGA, and 170 FGR cases and 273 non-cases at 36 wkGA.

a Adjusting for maternal height, maternal age, maternal body mass index, marital status, maternal age at stopping full-time education and deprivation.

b Adjusting for the same covariates as Model A plus objective smoking status.

c Adjusting for the same covariates as Model A plus self-reported smoking status.

d Odds ratio (95% CI) for one category increase in the paraxanthine tertile.

e Odds ratio (95% CI) when paraxanthine Tertile 1 is used as a reference group.

## Discussion

In this study, we used objective measures of smoking and caffeine intake during pregnancy and assessed their association with adverse pregnancy outcomes. We demonstrated stronger associations between smoking and both sPTB and FGR than other studies,^1^^,^^2^ though this only held true for women who had consistent exposure to smoking throughout pregnancy. Unlike previous reports, we found no evidence of a reduced risk of PE among smokers. On the other hand, consistently high levels of paraxanthine—representing high caffeine intake throughout pregnancy—were not associated with any studied outcome after accounting for smoking status using an objective measure. These data usefully inform clinicians and women on outcomes after smoking and caffeine consumption in pregnancy.

In a cohort that was relatively highly educated and generally lived in areas with low socio-economic deprivation, we observed that almost one in three women with consistent exposure to smoking throughout pregnancy did not self-report as smokers. Others have shown that reliance on self-reported data underestimates true smoking rates in pregnancy,^12^ particularly in settings where smoking cessation is encouraged.^29^ This discrepancy between self-reported and objective data may influence published association estimates between smoking and pregnancy complications. When smoking status was objectively quantified and classified, we observed a stronger association between exposure and adverse outcomes compared with previous literature. In particular, consistent smoking throughout pregnancy nearly tripled the risk of sPTB, more than quadrupled the risk of FGR and reduced birthweight by nearly 400 grams or by a median of 24%iles. These estimates are stronger than those found in meta-analyses [pooled odds ratio (OR) = 1.27, 95% CI: 1.21 to 1.33 for sPTB^2^; pooled OR = 1.65, 95% CI: 1.38 to 1.90 for FGR^1^]. The strength of the associations observed in the POP study likely relates to the classification of smoking status objectively with serial blood samples across the duration of pregnancy, capturing women who do not accurately self-report their smoking status or women who are consistently exposed to passive smoke. They may also relate to, for example, our use of a precise phenotype of FGR. Other studies tend to include all babies with a birthweight below the 10th centile in their definition of FGR,^10^^,^^11^ which includes a large proportion of healthy constitutionally small babies. Misclassification bias, occurring through reliance on self-reported data and over-inclusive definitions, will tend to dilute the strength of associations between smoking and perinatal outcome. However, we cannot exclude the possibility of other risk factors, such as maternal morbidities, maternal mental health and medication or drug exposures, that may be confounding the relationship between smoking and our measured outcomes.

Despite evidence that smoking may paradoxically reduce the risk of PE,^5^ with some reporting a ≤50% reduction,^30^ we did not observe any association. However, our study cannot definitively rule out a protective effect of smoking given that our 95% CIs were wide and included the point estimate from published meta-analyses (summarized relative risk = 0.67, 95% CI: 0.60 to 0.75^5^; pooled OR = 0.65, 95% CI: 0.58 to 0.73^31^; pooled OR = 0.51, 95% CI: 0.38 to 0.64^32^). We further explored this by examining the effects of smoking on pro- and anti-angiogenic growth factors that are suspected of underlying the pathogenesis of PE. It has been hypothesized that tobacco consumption products lower circulating concentrations of sFlt-1 and increase circulating concentrations of PlGF, therefore improving the overall angiogenic milieu that is normally disrupted by PE.^33^ We did not find any difference in sFlt-1 levels in women who smoked except in late pregnancy where sFlt-1 was higher—not lower—among those with consistent smoking exposure. There was also a progressive, graded increase in the adjusted difference in sFlt-1 levels with advancing gestation between women who were and were not exposed to smoking. On the other hand, maternal serum PlGF levels were >1 SD higher in early and mid-pregnancy in women with evidence of consistent exposure to smoking—but not those with inconsistent exposure—compared with those with none, which may support a more pro-angiogenic environment. Another observation was that, at early gestations, smoking appeared to primarily be associated with differences in maternal PlGF levels but not with maternal sFlt-1 levels whereas, at later gestations, this relationship reversed. Similar findings have been observed elsewhere^27^ and whereas we cannot comment on whether this observation adds any weight to the claims that smoking reduces the risk of PE, our observation highlights the importance of adjusting for smoking status when determining PlGF MoMs in combined first-trimester algorithms to prevent falsely reassuring results. This is recognized by the Fetal Medicine Foundation.^34^

In contrast to smoking, consistently high levels of paraxanthine throughout pregnancy—representing high caffeine intake—were not associated with any of the studied outcomes. Our findings are consistent with other studies which have shown that caffeine intake was not associated with increased risk of PE or sPTB.^35^^,^^36^ A possible protective effect was observed as those with moderate paraxanthine levels throughout pregnancy had a lower risk of GDM compared with those with low levels. Whereas it is difficult to separate the effects of diet, body mass index and caffeine intake on this risk given that caffeine is found in a wide array of foods and drinks, the potential benefit of caffeine consumption in reducing the risk of GDM has been reported previously.^37^^,^^38^ There have also been some reports that caffeine is associated with an increased risk of FGR^10^^,^^11^ and several meta-analyses have demonstrated an increased risk of lower birthweights.^3^^,^^4^ In our study, we did not observe any such risk after multivariable adjustment that included an objective measure of smoking. We hypothesized that this is because there is a strong correlation between paraxanthine and smoking, that has been recognized elsewhere^26^ and that was more fully controlled for in our regression models by using an objective measure to assess smoking. Supporting this hypothesis are studies that have found that the association between caffeine and FGR only existed among smokers.^11^^,^^39^ Indeed, smokers tend to consume more caffeine,^39^^,^^40^ metabolize caffeine at a faster rate by inducing cytochrome P450 1A2,^41^ the principal enzyme involved in caffeine metabolism, and therefore have lower serum caffeine but higher serum paraxanthine concentrations^39^^,^^42^ when compared with non-smokers. Whereas this interaction of smoking on caffeine metabolism in pregnancy is complex and beyond the scope of the present study, we were able to show that, when paraxanthine tertiles were examined at different time points, there was an increased association between the highest tertile of paraxanthine and FGR in univariable analyses that disappeared after adjusting for maternal characteristics and objective smoking status but not always self-reported smoking status. This highlights that there is value in using an objective measure to assess smoking in pregnancy to prevent smoking from confounding the associations between caffeine intake and adverse outcomes.^26^

The main strength of this study was our ability to assess smoking and caffeine exposure objectively and at multiple time points during pregnancy. This prevented recall bias, allowed capturing of women who had passive exposure to smoke or were unaware of caffeine ingestion and provided a more accurate reflection of women’s intake across the course of pregnancy rather than at a single time point. However, there were some limitations. Given that our objective measures were based on relative concentrations of serum metabolites (i.e. MoM), we could not extrapolate to the type of smoking exposure (e.g. active, passive or previous smoking) or quantify the amount of caffeine consumed by women. This limits comparison to other studies because MoM cannot be converted to a more universal unit (e.g. ng/mL). Women who had some (inconsistent) exposure to smoking were likely a heterogenous group that consisted of active, passive and previous smokers. Similarly women who had moderate levels of paraxanthine throughout pregnancy would have had more variable caffeine intake throughout pregnancy. Despite this heterogeneity, we did not further categorize women in these groups because group sizes became too small. We also did not use cut-offs to categorize consumption as our measurements were in MoM and any cut-off point was at risk of misclassification.^14^ Blood samples were taken at four discrete time points throughout pregnancy and therefore only provided a snapshot of the exposure in a given pregnancy unlike self-reported data. Given the half-life and metabolism of the metabolites, it was possible for women to have undetectable or high levels of metabolite depending on the time of their last cigarette or caffeine ingestion prior to blood draw. We did not have data related to the time of last cigarette or caffeine ingestion.

## Conclusion

Using objective measures of smoking and caffeine intake, we showed that consistent exposure to smoking throughout pregnancy was strongly associated with sPTB and FGR, whereas caffeine consumption was not associated with any adverse outcomes or with lower birthweight (absolute or percentile).

## Ethics approval

Cambridgeshire 2 Research Ethics Committee gave ethical approval for the study (reference number 07/H0308/163) and all participants provided written informed consent.

## Supplementary Material

dyad123_Supplementary_Data

## Data Availability

For the POP study, restrictions apply to the availability of data generated or analysed during this study to preserve patient confidentiality or because they were used under licence. The corresponding author will on request detail the restrictions and any conditions under which access to some data may be provided. Data requests on the POP study data can be made to the corresponding author.
