# Supplementary material for: Objective measures of smoking and caffeine intake and the risk of adverse pregnancy outcomes
Source: Int J Epidemiol. 2023 Sep 28;52(6):1756–65. doi: 10.1093/ije/dyad123 (PMC10749751; doi:10.1093/ije/dyad123)
Supplement: dyad123_Supplementary_Data [file dyad123_supplementary_data.docx]

**Supporting information**

1. **Supplementary Figure S1.** Flow diagram of case cohort design in the POP study.
2. **Supplementary Table S1.** Number and proportion of women with detectable cotinine levels at each time point.
3. **Supplementary Table S2.** Association between objective smoking status and adverse pregnancy outcomes, adding weight gain during pregnancy to the multivariable models.
4. **Supplementary Table S3.** Association between objective caffeine exposure and adverse pregnancy outcomes, adding weight gain during pregnancy to the multivariable models.

POP study cohort

n = 4212 completed

n = 277 without any of the studied adverse outcomes

n = 592

n = 46

Random subcohort selection

n = 325

n = 323

n = 2 with three missing samples ^b^

Cases with at least one of the studied adverse outcomes ^a^

n = 644

n = 638

n = 6 with three missing samples ^b^

**Supplementary Figure S1.** Flow diagram of case cohort design in the POP study.

POP = Pregnancy Outcome Prediction

^a^ Cases are women who had at least one studied adverse outcome: PE, sPTB, FGR, or GDM.

^b^ Women with only one of the four possible blood samples at 12, 20, 28, and 36 wkGA were excluded (n=8).

^c^ For the caffeine analyses, the total population studied was n=915. For the smoking analyses, women who self-reported using nicotine replacement therapy were excluded (n=1) and so the total population studied was n=914. This single case was in the random subcohort and did not have any of the studied adverse outcomes.

^d^ Of the 277 women in the random subcohort who did not have any of the studied adverse outcomes, n=220 had no missing samples, n=51 had one missing sample, and n=6 had two missing samples. Of the 46 women in the random subcohort who had at least one of the studied adverse outcomes, n=29 had no missing samples, n=13 had one missing sample, and n=4 had two missing samples. Of the 592 women not in the random subcohort who had at least one of the studied adverse outcomes: n=268 had no missing samples, n=260 had one missing sample, and n=64 had two missing samples.

**Supplementary Table S1.** Number and proportion of women with detectable cotinine levels at each time point.

|  | **0 missing samples** | **1 missing sample** | **2 missing samples** |
| --- | --- | --- | --- |
| **12 wkGA** ^a,b^ | 79 / 517 (15.3) | 40 / 298 (13.4) | 18 / 57 (31.6) |
| **20 wkGA** ^a,b^ | 74 / 517 (14.3) | 33 / 303 (10.9) | 19 / 51 (37.3) |
| **28 wkGA** ^a,b^ | 73 / 517 (14.1) | 29 / 303 (9.6) | 10 / 32 (31.3) |
| **36 wkGA** ^a,b^ | 67 / 517 (13.0) | 8 / 65 (12.3) | 0 / 8 (0.0) |

^a^ Data are expressed as n / N (%) where n represents the number of women with detectable cotinine levels at the given time point and N represents the number of women with the given number of missing samples at the given time point.

^b^ Percentages are unweighted and do not account for the case-cohort design and so are not representative of whole Pregnancy Outcome Prediction study.

**Supplementary Table S2.** Association between objective smoking status and adverse pregnancy outcomes, adding weight gain during pregnancy to the multivariable models.

|  | **Objective smoking status** | |
| --- | --- | --- |
|  | **Some exposure to smoking throughout pregnancy** | **Consistent exposure to smoking throughout pregnancy** |
|  | **Multivariable analyses** ^a,e^ | |
|  | aOR (95% CI) | |
| **PE** | 1.42 (0.78, 2.58)  *P* = 0.25 | 1.10 (0.48, 2.53)  *P* = 0.83 |
| **sPTB** | 1.02 (0.49, 2.16)  *P* = 0.95 | 2.59 (1.14, 5.88)  *P* = 0.02 |
| **FGR** | 1.40 (0.78, 2.50)  *P* = 0.26 | 3.94 (2.05, 7.55)  *P* < 0.001 |
| **GDM** | 1.32 (0.72, 2.44)  *P* = 0.37 | 1.06 (0.46, 2.42)  *P* = 0.89 |
|  | β coefficient (95% CI) | |
| **Birthweight (grams)** ^b^ | -140 (-262, -18)  *P* = 0.02 | -353 (-587, -120)  *P* = 0.003 |
| **Birthweight Z-score** ^b,c^ | -0.23 (-0.46, 0.00)  *P* = 0.05 | -0.53 (-0.85, -0.22)  *P* = 0.001 |
| **Birthweight percentile ^b,^**^d^ | -7 (-15, 2)  *P* = 0.11 | -20 (-35, -5)  *P* = 0.007 |

The numbers of cases and non-cases from the random subcohort included in the analyses of the binary outcomes were 190 PE cases and 302 non-cases, 111 sPTB cases and 310 non-cases, 215 FGR cases and 301 non-cases, and 185 GDM cases and 311 non-cases.

PE = preeclampsia, sPTB = spontaneous preterm birth, FGR = fetal growth restriction, GDM = gestational diabetes mellitus.

^a^ Reference group is women with no exposure to smoking throughout pregnancy.

^b^ For birthweight regression analyses, the entire case cohort (n=914) was studied by weighting the non-cases of the random subcohort by the inverse of the sampling fraction.

^c^ For birthweight Z-score, β coefficients from linear regression analyses were given per one standard deviation increase in birthweight percentile.

^d^ For birthweight percentile, quantile regression analyses using the median were performed.

^e^ Adjusting for maternal height, maternal age, maternal body mass index, marital status, maternal age at stopping full time education, deprivation, and weight gain during pregnancy.

**Supplementary Table S3.** Association between objective caffeine exposure and adverse pregnancy outcomes, adding weight gain during pregnancy to the multivariable models.

|  | **Objective caffeine exposure** | |
| --- | --- | --- |
|  | **Moderate levels of paraxanthine throughout pregnancy** | **High levels of paraxanthine throughout pregnancy** |
|  | **Multivariable analyses** ^a,e^ | |
|  | aOR (95% CI) | |
| **PE** | 0.79 (0.43, 1.44)  *P* = 0.43 | 0.90 (0.41, 1.99)  *P* = 0.79 |
| **sPTB** | 0.83 (0.40, 1.70)  *P* = 0.61 | 0.83 (0.33, 2.10)  *P* = 0.69 |
| **FGR** | 0.89 (0.48, 1.66)  *P* = 0.71 | 1.15 (0.53, 2.51)  *P* = 0.73 |
| **GDM** | 0.56 (0.32, 0.98)  *P* = 0.04 | 0.59 (0.27, 1.29)  *P* = 0.18 |
|  | β coefficient (95% CI) | |
| **Birthweight (grams)** ^b^ | -27 (-174, 120)  *P* = 0.72 | 12 (-206, 230)  *P* = 0.91 |
| **Birthweight Z-score** ^b,c^ | -0.16 (-0.44, 0.13)  *P* = 0.28 | -0.05 (-0.41, 0.32)  *P* = 0.80 |
| **Birthweight percentile ^b,^**^d^ | -6 (-17, 5)  *P* = 0.31 | 4 (-10, 18)  *P* = 0.54 |

The numbers of cases and non-cases from the random subcohort included in the analyses of the binary outcomes were 190 PE cases and 303 non-cases, 111 sPTB cases and 311 non-cases, 215 FGR cases and 301 non-cases, and 185 GDM cases and 312 non-cases.

PE = preeclampsia, sPTB = spontaneous preterm birth, FGR = fetal growth restriction, GDM = gestational diabetes mellitus.

^a^ Reference group is women with low levels of paraxanthine throughout pregnancy.

^b^ For birthweight regression analyses, the entire case cohort (n=915) was studied by weighting the non-cases of the random subcohort by the inverse of the sampling fraction.

^c^ For birthweight Z-score, β coefficients from linear regression analyses were given per one standard deviation increase in birthweight percentile.

^d^ For birthweight percentile, quantile regression analyses using the median were performed.

^e^ Adjusting for objective smoking status, maternal height, maternal age, maternal body mass index, marital status, maternal age at stopping full time education, deprivation, and weight gain during pregnancy.
